# Supplementary material for: Population cycles and species diversity in dynamic Kill-the-Winner model of microbial ecosystems
Source: Sci Rep. 2017 Jan 4;7:39642. doi: 10.1038/srep39642 (PMC5209715; doi:10.1038/srep39642)
Supplement: Supplementary Information [file srep39642-s1.pdf]

Supplementary materials for: Population cycles and species diversity in dynamic Kill-the-Winner model of microbial ecosystems

Sergei Maslov*

Department of Bioengineering,

and Carl R. Woese Institute for Genomic Biology,

University of Illinois,

Urbana-Champaign, IL 61801, USA

Kim Sneppen†

Center for Models of Life, Niels Bohr Institute,

University of Copenhagen,

2100 Copenhagen, Denmark

(Dated: November 10, 2016)

* maslov@illinois.edu

† ksneppen@gmail.com

SUPPLEMENTARY MATERIALS

Diversity for an arbitrary fitness distribution

Let's consider the case where growth rates g_i is selected from probability distribution with standard deviation σ: $P(g) = (1/\sigma)f(g/\sigma)$. Here $f(x)$ is the PDF of the distribution with standard deviation 1. For a large initial population N the sum $\sum_{g_i > g_{cc}}(g_i - g_{cc})$ in eq. 3 can be approximated with the integral:

$$\frac{N\Delta t}{|\log\gamma|}\int_{g_{cc}}^{\infty}(g-g_{cc})f\left(\frac{g}{\sigma}\right)\frac{dg}{\sigma} = \\ = \frac{N\sigma\Delta t}{|\log\gamma|}\int_{y_{cc}}^{\infty}(y-y_{cc})f(y)dy = 1 \tag{S1}$$

where $y_{cc} = g_{cc}/\sigma$ is normalized minimal growth rate needed for a long time survival of the species. The diversity of surviving species is then given by

$$D = N\int_{y_{cc}}^{\infty} f(y)dy \tag{S2}$$

For exponential distribution , $f(y) = exp(-y)$, one gets

$$N\sigma\delta t \exp\left(-\frac{g_{cc}}{\sigma}\right) = |\log\gamma| \tag{S3}$$

$$D = N\sigma \exp\left(-\frac{g_{cc}}{\sigma}\right) \tag{S4}$$

or

$$D = \frac{|\log\gamma|}{\sigma\delta t} \quad .$$

In the case of the Gaussian distribution, $f(y) = \exp(-\frac{y^2}{2})\frac{1}{\sqrt{2\pi}}$, Eqs. S1, S2 cannot be solved analytically. Numerical solution shown as dashed line in Fig. 1b closely resembles simulations.

Diversity with variable collapse ratios

The model can be directly generalized to the case where different species have different collapse ratios γ_i. This may for example be the case for bacteria with different degrees of vulnerability to phages, or bacteria with different ways to partition their population in

physical space. The modified Eqs. reads

$$c_i = \frac{(g_i - g_{cc})}{|\log \gamma_i|} \tag{S5}$$

$$1 = \sum_{g_i > g_{cc}} \frac{(g_i - g_{cc})}{|\log \gamma_i|} \tag{S6}$$

where the first equation again imply that only the $g_i > g_{cc}$ will contribute.

To solve eq. S6 the sum in eq. S6 is successively tested for species that is rank ordered from the largest values of $g_i = g_1$, until a value $D = i$ where it provide a solution for a $g_{cc} \in [g_{D+1}, g_D]$.

Allowing individual species to have different γ_i only moderately changes the diversity D compared to the uniform case of $\gamma_i = \gamma$ (see Fig. S1). We tested several variants in which γ_i was assigned to individual species as described by Eq. S6 or in which γ_i was randomly varying between collapse events. Allowing γ_i to vary between species or collapse events also did not affect the distributions of populations sizes (data not shown).

Kill-the-King model

For convenience we choose an arbitrary time point $t = 0$ and reorder the populations in the order of decreasing sizes so that $P_1(0)$ corresponds to the largest population, $P_2(0)$ - to the second largest, and so on with $P_N(0)$ being the smallest population. The new cycle starts with $P_1(0)$ which collapses down to γ while the rest of the species remain unchanged with the total population of $1 - P_1(0)$. After the collapse all species grow "instantly" to the carrying capacity resulting in $P_1(1) = \gamma/(1 - P_1(0) + \gamma)$.

At the subsequent phage attack the second population $P_2(1)$ collapses which after rescaling leaves the first two populations as respectively

$$P_1(2) = \gamma/(1 - P_1(0) + \gamma)/(1 - P_2(1) + \gamma) \;\; and$$
$$P_2(2) = \gamma/(1 - P_2(1) + \gamma)$$

The subsequent $N - 2$ collapses of populations 3 to N do not change the ratio between the first two populations, implying that the updated ratio between the second and the first population remains $P_2(N)/P_1(N) = P_2(2)/P_1(2) = 1 - P_1(0) + \gamma$.

As derived above then $\delta_1(t + N) = 1 - P_1(t) + \gamma$. Taking into account that $1 = \sum_i P_i = P_1 + P_1\delta_1 + P_1\delta_1\delta_2 + \ldots P_1\delta_1\delta_2 \ldots \delta_{N-1}$, in the limit where all $\delta_i \ll 1$ up to the second order in

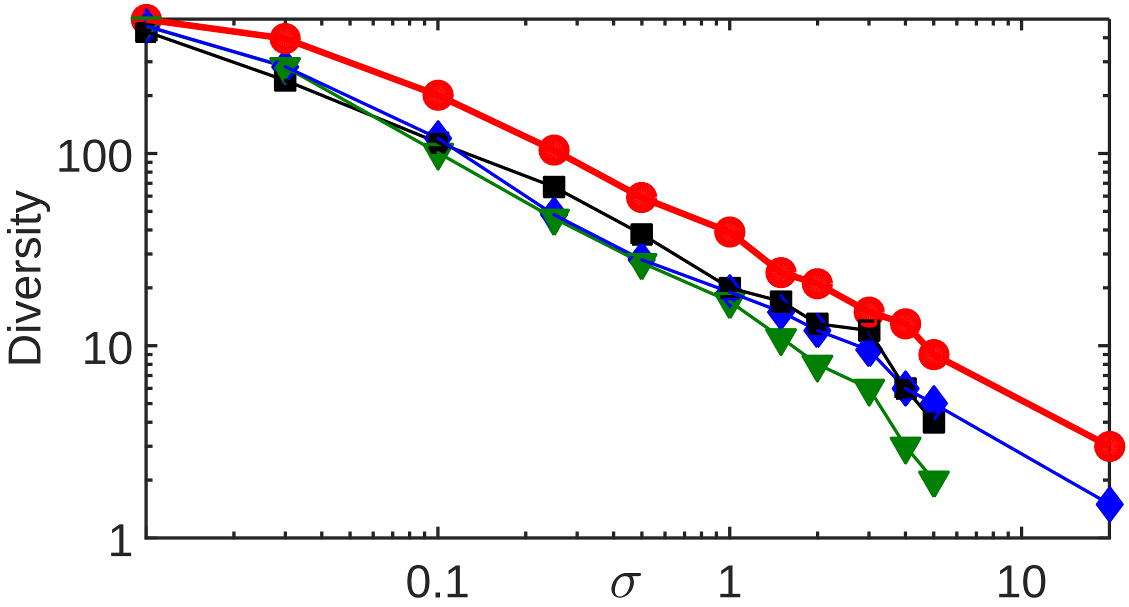

FIG. S1. *Final diversity D counted as number of species with populations* $> 10^{-20}$ *as the function of spread* σ *of initially assigned growth rates among* $N = 500$ *species. The red curve marked with circles corresponds to the KtW model with fixed* $\gamma = 10^{-6}$, *while the blue curve marked with diamonds - to fixed* $\gamma = 10^{-3}$. *Te black curve marked with squares show simulation results when* γ_i *varies between species (quenched noise), while the red curve marked with triangles - when it varies between collapse events (annealed noise). In these two cases* $\log_{10} \gamma_i$ *was drawn from a uniform distribution between -1 and -5 so that its geometric average of* 10^{-3} *corresponds to the blue curve.*

δ_i one gets $P_1 \simeq 1/(1+\delta_1+\delta_1\delta_2)$ or $1-P_1 \simeq (\delta_1+\delta_1\delta_2)/(1+\delta_1+\delta_1\delta_2) \simeq (\delta_1+\delta_1\delta_2)\cdot(1-\delta_1) \simeq \delta_1 + \delta_1(\delta_2 - \delta_1)$. Thus the following equation describes change of δ after one full round of population collapses: $\delta_1(t+N) = 1 - P_1(t) + \gamma \simeq \delta_1(t) + \delta_1(t)(\delta_2(t) - \delta_1(t))$ up to $O(\delta^2)$. Since in the course of one cycle of collapses each population in turn becomes the largest one, the above equation for δ_1 applies to i other than 1:

$$\delta_i(t+N) - \delta_i(t) = \delta_i(t) \cdot (\delta_{i+1}(t) - \delta_i(t)) \quad . \tag{S7}$$

In the long time limit the populations in the KtK model asymptotically approach an equidistant distribution on the logarithmic scale. Thus $\delta_i(\infty) = \delta^* = \gamma^{1/N}$. Uniform distribution of population sizes on the logarithmic scale corresponds to the power law species abundance distribution $P(S) \sim S^{-1}$.

In the continuous limit in time t and space $x = i$ the Eq. S7 can be rewritten taking into account $\delta_i(t+N) - \delta_i(t) \simeq N\partial\delta(x,t)/\partial t$, while $\delta_{i+1}(t) - \delta_i(t) \simeq \partial\delta(x,t)/\partial x + (1/2)\partial^2\delta(x,t)/(\partial x)^2$. Note the appearance of the second derivative over x due to the fact that $\delta_{i+1}(t) - \delta_i(t)$ is centered half-way between i and $i+1$ and thus is shifted up from i by 1/2. Thus the gap dynamics in our model is described by:

$$\frac{N\partial\delta}{\partial t} = \delta \cdot \frac{\partial\delta}{\partial x} + \frac{\delta}{2} \cdot \frac{\partial^2\delta}{\partial x^2} \tag{S8}$$

Compared to the traditional Burgers equation the diffusion coefficient is not constant but proportional to δ.

In KtW model the population collapses do not always happen in the order dictated by their relative sizes. When a collapse of a smaller population happens it only causes a small rescaling of populations, and the subsequent collapse of the largest population leads to a situation where these two populations are nearly equal in size. This dramatically increase the likelihood for further re-orderings between these two species, resulting in an intermittent dynamical period of fights for "dominance" between these two species.

The most likely "mistake" changing the order of collapses is when the second largest population " jumps the gun"and collapses ahead of the largest one. Repeating the above derivation in this case one gets $P_2(2)/P_1(2) = 1 - P_2(0) + \gamma \simeq 1 - P_2(0)$. In the asymptotic case where populations are equidistantly distributed on the logarithmic scale one has $P_k(\infty) = \gamma^{(k-1)/N} \cdot (1-\gamma^{1/N})/(1-\gamma)$. In the limit where $\gamma^{1/N} = \delta^* \ll 1$ the top two populations occupy most of the carrying capacity and are approximately equal to $1 - \delta^*$ and δ^* (up to the second order in δ^*). Hence, when the second largest population collapses ahead of the first one it leads to an instant and dramatic increase in δ_1 to $1 - P_2(0) = 1 - \delta^*$ up from its steady state value of δ^*. The Eq. S7 describes the subsequent relaxation of $\delta_1(t)$ back to δ^*. Indeed, if one disregards the first term in the r.h.s. of the equation but $\delta_1 \simeq 1$ one gets $d\delta_1/d(t/N) = -\delta_1^2$. Hence initially the gaps starts relaxing as

$$\delta_1(t) = \frac{1-\delta^*}{t/N} \tag{S9}$$

It takes about $(1-\delta^*)/\delta^*$ collapses for $\delta_1(t)$ to get down close to δ^*. At this point one cannot completely disregard δ_2 but one can still assume that it stays close to its the steady state

value $\delta^* = \gamma^{1/N}$. In this case $d\delta_1/d(t/N) = \delta^*\delta_1 - \delta_1^2$ or

$$
\begin{aligned}
d\delta_1 \;\cdot\; (-1/\delta_1 + 1/(\delta_1 - \delta^*)) &= -\delta^* d(t/N) \\
\delta_1(t) &= \frac{\delta^*}{1 - \frac{1-2\delta^*}{1-\delta^*}\exp(-\delta^* t/N)}.
\end{aligned}
\tag{S10}
$$
